# Toll-Like Receptors, Associated Biological Roles, and Signaling Networks in Non-Mammals

**DOI:** 10.3389/fimmu.2018.01523

**Published:** 2018-07-02

**Authors:** Li Nie, Shi-Yu Cai, Jian-Zhong Shao, Jiong Chen

**Affiliations:** ^1^Laboratory of Biochemistry and Molecular Biology, School of Marine Sciences, Ningbo University, Ningbo, China; ^2^College of Life Sciences, Zhejiang University, Hangzhou, China

**Keywords:** toll-like receptor, signaling pathway, myeloid differentiation primary response protein 88, TRIF, TICAM2, non-mammalian, evolution

## Abstract

The innate immune system is the first line of defense against pathogens, which is initiated by the recognition of pathogen-associated molecular patterns (PAMPs) and endogenous damage-associated molecular patterns (DAMPs) by pattern recognition receptors (PRRs). Among all the PRRs identified, the toll-like receptors (TLRs) are the most ancient class, with the most extensive spectrum of pathogen recognition. Since the first discovery of Toll in *Drosophila melanogaster*, numerous TLRs have been identified across a wide range of invertebrate and vertebrate species. It seems that TLRs, the signaling pathways that they initiate, or related adaptor proteins are essentially conserved in a wide variety of organisms, from Porifera to mammals. Molecular structure analysis indicates that most TLR homologs share similar domain patterns and that some vital participants of TLR signaling co-evolved with TLRs themselves. However, functional specification and emergence of new signaling pathways, as well as adaptors, did occur during evolution. In addition, ambiguities and gaps in knowledge still exist regarding the TLR network, especially in lower organisms. Hence, a systematic review from the comparative angle regarding this tremendous signaling system and the scenario of evolutionary pattern across Animalia is needed. In the current review, we present overview and possible evolutionary patterns of TLRs in non-mammals, hoping that this will provide clues for further investigations in this field.

## Introduction

Toll-like receptors (TLRs) are a class of pattern recognition receptors (PRRs) that initiate the innate immune response by sensing conserved molecular patterns for early immune recognition of a pathogen (1). The typical TLRs are type I transmembrane proteins that contain three structural domains: a leucine-rich repeats (LRRs) motif, a transmembrane domain, and a cytoplasmic Toll/IL-1 receptor (TIR) domain. The LRRs motif is responsible for pathogen recognition, whereas the TIR domain interacts with signal transduction adaptors and initiates signaling (2). Since the first report of a Toll protein in the fruit fly *Drosophila melanogaster* (3), 10 TLRs have been identified in human (TLR1–TLR10) and 13 in mouse (TLR1–TLR13). It appears that most mammalian species share a similar repertoire of TLR homologs, although with exceptions (4, 5). For instance, a gene coding for the human TLR10 homolog is also present in mouse, but appears to have been modified by a retrovirus in the latter (6). Moreover, TLR11–TLR13 are expressed in mouse, whereas they are absent in human (7). Molecules broadly shared by pathogens, known as pathogen-associated molecular patterns (PAMPs), and the host endogenous damage-associated molecular pattern molecules (DAMPs), can be recognized by TLRs (8–11). This kind of recognition is multifarious, depending on the type of TLR. For example, mammalian TLR4 is responsible for detecting lipopolysaccharide (LPS), the component of Gram-negative (G^−^) bacteria, whereas murine TLR13 recognizes bacterial 23S rRNA (8).

Toll-like receptors are essential molecular receptors through which the immune system "senses" the risk to protect the host from pathogenic microorganisms or endogenous threats (12). Numerous roles of TLRs have been identified, such as the recognition of self and non-self antigens; detection of invading pathogens; bridging the innate and adaptive immunity; and regulation of cytokine production, proliferation, and survival (8–11). TLR functions are mediated by subsequently initiated signaling pathways, resulting in the production of various cytokines and chemokines. Currently, TLR signaling pathways are classified into two distinct types, namely, the myeloid differentiation primary response protein 88 (MyD88)-dependent pathways and the TIR domain-containing adaptor-inducing IFNβ (TRIF)-dependent pathways (13). The MyD88-dependent response is utilized by almost all the TLRs, with the exception of TLR3. Upon ligand recognition and TLR dimerization, MyD88 binds to the TIR domain of the corresponding TLR through homotypic/heterotypic interactions. Subsequently, IL-1 receptor-associated kinase 4 (IRAK4) is recruited through the death domain of MyD88, leading to the formation of a Myddosome complex (14), and autophosphorylation of IL-1 receptor-associated kinase 1 (IRAK1). Afterward, the protein tumor necrosis factor (TNF) receptor-associated factor 6 (TRAF6) is activated, which in turn activates the TAK1/TGF-β-activated kinase (TAB) complex through K-63-linked polyubiquitination of TAK1 and TRAF6 (15). This is followed by IκB kinase (IKK)-mediated phosphorylation and degradation of I kappa B alpha (IκBα). Degradation of this inhibitor finally leads to nuclear translocation of the transcription factor NF-κB (16), which induces the transcription of genes encoding inflammatory cytokines.

Generally, the TRIF-dependent pathway is considered to be specific for only few TLRs, such as TLR3 and TLR4 in mammals. Transcription factors, including NF-κB, activating protein-1 (AP-1), and interferon (IFN) regulatory factor (IRF) family members, can be activated by the TRIF-dependent pathway, collectively inducing the production of pro-inflammatory cytokines and/or type I IFN (IFN1) (17). TLR3 is activated by recognizing double-stranded RNA (dsRNA), which is followed by the recruitment of TRIF. TRIF activates TANK-binding kinase 1 (TBK1) and receptor-interacting serine/threonine kinase 1 (RIPK1), respectively, which creates a branch in the signaling pathway. The TRIF/TBK1 signaling complex phosphorylates IRF3, allowing its translocation to the nucleus and the production of IFN1. Activation of RIPK1 causes a series of signal transduction events in the same manner as the MyD88-dependent pathway (18). TLR4 functions as an LPS receptor in mammals, and the TLR4-myeloid differentiation protein 2 (MD2)-LPS complex activates early-phase NF-κB and mitogen-activated protein kinase (MAPK) after the recruitment of MyD88 and MyD88-adapter-like (MAL) adaptors. After the TLR4-MD2–LPS complex enters the cell *via* endocytosis, it interacts with the TRIF and TIR domain-containing adapter molecule 2 (TICAM2, also known as TRAM) adaptors. This TRIF-dependent pathway not only induces the production of IFN1 but also activates IRF7 and late-phase NF-κB (19). Ultimately, the TLR signaling leads to the induction or suppression of genes that fine-tune the inflammatory response.

In addition to the extensively studied molecular forms and biological roles of TLRs in mammals, recent genome surveys of various organisms revealed that TLRs or their related proteins are also present in an extensive range of non-mammalian species, and that these organisms harbor TLRs that are distinct from those of mammals. Examples include TLR15 in chicken, TLR24 in lamprey, and TLR28 in miiuy croaker (20–23). The aim of the current review was to present a detailed description of the roles of TLRs in non-mammals. Further, based on the current knowledge, we also suggest possible scenarios for the evolution of these proteins from invertebrates to mammals.

## TLRs in Invertebrates

The number and types of TLR family members in invertebrates varies among species, ranging from one to hundreds of members. For example, the nematode *Caenorhabditis elegans* harbors only one Toll-encoding gene, whereas the echinoderm *Strongylocentrotus purpuratus* has an expanded repertoire to 222 TLR-encoding genes. Based on the number of CF motifs (cysteine clusters at the C-terminal end of LRRs, LRRCT), the TLRs can be divided into two basic categories, the protostome-type (P-type, also known as mccTLR) and the vertebrate-type (V-type, also known as sccTLR) (24). P-type TLRs have a single cysteine cluster at LRRCT, while the V-type have multiple cycteine clusters at LRRCT, and sometimes even at the N-terminal end (LRRNT). The P-type TLRs have only been identified in invertebrates, which may represent an ancient form of TLR. On the other hand, all the vertebrate TLRs and some invertebrate TLRs belong to V-type (25). It has been suggested that P-type TLRs do not directly bind PAMPs, unlike the vertebrate V-type TLRs, as has been demonstrated by the most well characterized P-type TLR, *Drosophila* Toll-1 (3). The invertebrate phyla in which most TLRs have been identified include the Porifera, Coelenterata, Platyhelminthes, Nematoda, Annelida, Echinodermata, Mollusca, and Arthropoda, which will be described below in detail.

### Porifera

At present, details on poriferan TLRs have mainly been reported in *Amphimedon queenslandica* and *Suberites domuncula*. An LRR domain-containing protein with Ig- and epidermal growth factor (EGF)-like domains, and two TIR domain-containing proteins with N-terminal IL-1R-like Ig domains have been identified in *A. queenslandica* (26–28). Similarly, a TIR only protein (Sd-TLR) with a transmembrane domain has been identified in the sponge *S. domuncula*, although no LRR domain-containing proteins have been detected in this species (29).

Myeloid differentiation primary response protein 88 and NF-κB homologs involved in the TLR-to-NF-κB pathway were found in *A. queenslandica* and *S. domuncula*, indicating that MyD88-mediated TLR signaling pathway has already appeared in the poriferans (26, 30, 31). Moreover, expressions of other adaptor proteins involved in this TLR-to-NF-κB pathway are observed during early development of *A. queenslandica*, revealing that this pathway is associated with development (26). It has been demonstrated that Sd-TLR continuously interacts with microbes and may participate in the immune regulation of *S. domuncula* (29, 32). The expression of a caspase-like protease is detected when *S. domuncula* is treated with a synthetic lipoprotein mimic [Pam3Cys-Ser-(Lys)4], and this protease is likely related to the apoptotic process during pathogen infection (29). Furthermore, this kind of protease induction is abolished upon pre-incubation of the mimic with Sd-TLR, indicating that Sd-TLR is able to sense PAMPs and activate a pro-apoptotic pathway (29). However, the mechanism underpinning the interaction between Sd-TLR and the lipoprotein mimic remains unclear, as Sd-TLR lacks an LRR domain that is required for PAMP binding (29). The anti-pathogen pathway of *S. domuncula* is probably MyD88-dependent, as LPS treatment induces the expression of MyD88 (32).

### Cnidaria

The phylum Cnidaria is a morphologically primitive outgroup to bilaterians and comprises approximately 10,000 aquatic organisms, including corals, *Hydra*, sea anemones, and jellyfish (33). Many proteins related to TLR-to-NF-κB pathway, but no canonical TLRs, were identified in *Hydra* species. In addition, two transmembrane TIR domain-containing proteins and two LRR domain-containing proteins have been identified in *Hydra* (34–36). A chimeric protein combining the human TIR domain with *Hydra* LRR protein (HyLRR-2) can activate the NF-κB reporter in HEK293 cells in response to flagellin but not LPS (34). Therefore, flagellin may be the ligand of HyLRR-2 initiating innate immune signaling. Studies on *Hydra* have also suggested a role for its canonical TLR pathway in pathogen defense, as silencing of a TIR domain-containing protein resulted in a loss of antimicrobial peptide production, and *Hydra* became more sensitive to *Pseudomonas aeruginosa* infection when MyD88 was silenced (34, 37).

Two TIR domain-only genes have been identified in the genome of the sea anemone *Exaiptasia pallida*; they may encode the same protein (36, 38). To date, no LRR-only proteins or canonical TLRs have been identified in *E. pallida* (36, 38), while a single P-type TLR has been identified in the sea anemone *Nematostella vectensis*. *N. vectensis* P-type TLR (Nv-TLR) can activate NF-κB signaling in human cells in response to *Vibrio coralliilyticus* infection and flagellin, indicating that the Nv-TLR-activated pathway may be important for pathogen detection and immune response (39). Furthermore, the interaction of human MAL and MyD88 with the intracellular TIR domain of Nv-TLR has also been demonstrated (39). These findings reveal that Nv-TLR can recognize bacterial flagellin and initiate a MyD88-mediated signaling pathway. In addition, Nv-TLR knockdown leads to an abnormal embryonic development of *N. vectensis*, suggesting that Nv-TLR may also participate in early development (39). Nevertheless, little is known about the specific downstream signaling pathway or the ligand responsible for Nv-TLR involvement in the developmental and immune processes (39, 40).

Recent transcriptomic studies revealed that canonical TLR members and components associated with NF-κB activation are expressed in several other cnidarians, including the corals *Acropora digitifera, Acropora millepora*, and *Orbicella faveolata* (41, 42). 1 V-type TLR, 3 P-type TLRs, and 13 TIR-only proteins were identified in the reef-building coral *A. digitifera* (36, 43). Similarly, 1 P-type TLR and 10 TIR domain-containing proteins were found in its close relative, *A. millepora* (36, 44). In addition, four TIR-only proteins and a single P-type TLR were identified in the stony coral *O. faveolata*. The expression of genes related to NF-κB signaling pathway can be induced after stimulation of *O. faveolata* tissue with LPS. Further, *O. faveolata* P-type TLR (Of-TLR) can interact with human MyD88 and activate downstream NF-κB signaling pathways, indicating that the function of *O. faveolata* TLR is conserved to its human homologs (45).

### Platyhelminthes

The roles of platyhelminth TLRs have mainly been explored in planarians, turbellarians, and rotifers. As non-parasitic flatworms, Planarians are of great evolutionary importance for the analysis of injury-induced immune responses and the regeneration process in metazoans (46, 47). In the fresh water planarian *Schmidtea mediterranea*, several proteins involved in TLR-to-NF-κB pathway have been identified, albeit the TLRs of this flatworm are not canonical, but TIR and LRR-only proteins (25, 48, 49). Upregulation of TLRs, MyD88, TRAF, and IRAK occurs during head regeneration of *S. mediterranea*, indicating the TLR-initiated signaling pathway likely plays a role in preventing infection during the regeneration process (48). Additionally, NF-κB expression is detected in its intestinal phagocytes, and NF-κB knockdown leads to the lysis of intestinal tissues and *S. mediterranea* dissociation, indicating that NF-κB plays a role in the intestinal function in this species (49). However, the exact mechanisms and underpinning signaling networks require further investigation. Although genes encoding TIR- and LRR-domain proteins have been identified in the genome of the rotifer *Adineta vaga*, no canonical TLRs have been found therein (50). Furthermore, immune responses or pathways in rotifers have not been reported. Hence, a comprehensive elucidation of the immune responses initiated by TLRs against pathogens, or PAMP stimulation and the mechanisms involved in the phylum Platyhelminthes should be conducted in the future.

### Nematoda

*Caenorhabditis elegans* is a classic model organism for the study of nematodes. It has been demonstrated that *C. elegans* expresses a TIR-domain containing protein, a canonical P-type TLR (TOL-1), and other components analogous to those of mammalian TLR signaling pathways (25, 51–57). Nevertheless, many components associated with the TLR-to-NF-κB signaling pathway are absent in *C. elegans*, including MyD88, IKK, and NF-κB, implying that, therefore, TOL-1 does not initiate the NF-κB-dependent signaling pathways (53, 57). Although previous studies have shown that TOL-1 is important for early development and pathogen recognition in *C. elegans*, the downstream pathways activated by TOL-1 during early development remain unclear (53, 57). Recently, Brandt and colleagues revealed that TOL-1 contributes to the development and function of BAG neurons required for the pathogen-avoidance behavior in *C. elegans via* TOL-1-to-p38 MAPK pathway (51, 58). In addition, *C. elegans* with a TOL-1 mutation is more susceptible to certain bacterial infections than the wild type, suggesting that TOL-1 also likely participates in microbial defense (53, 56, 59, 60).

Single P-type TLRs have also been identified in *Caenorhabditis briggsae, Caenorhabditis brenneri*, and *Caenorhabditis japonica* (51, 61–63). However, the associated-downstream pathways or functions of these TLRs have not been elucidated. Given that the sequences of TLRs among *Caenorhabditis* species are conserved (63), we speculate that the biological roles of these proteins are similar to those elucidated in *C. elegans*.

### Annelida

Davidson and colleagues presented preliminary evidence for the existence of TLRs in the genomes of annelids, including polychete worm *Capitella capitata* and leech *Helobdella robusta* (64). They identified 16 and 105 TLRs loci in the *H. robusta* and *C. capitata* genomes, respectively (64). Notably, the sequences of TLRs in *C. capitata* are very similar and may have arisen from recent gene duplications, which likely explains the large number of TLR-like genes present in its genome (64). Among those TLRs loci, 22 have been confirmed to encode V-type TLRs and 1 to encode P-type TLR in *C. capitata* (64), with 8 V-type TLR detected in *H. robusta* (65).

In *Hirudo medicinalis*, a TLR named Hm-TLR1 has been identified, which appears to be a chimeric combination of the intraendosomal domain of human TLR3 and the cytoplasmic section of TLR13. The expression of Hm-TLR1 is detected in microglial cells and neurons, and Hm-TLR1 has been suggested to play important roles in immunity (66, 67). Upregulation of Hm-TLR1 is observed in *H. medicinalis* nerve cords upon stimulation with several types of bacterial molecules, and also at lesion sites during the following neuroregeneration (66, 67). Another study has revealed that differentiated *H. medicinalis* neurons respond to LPS through a MyD88-dependent pathway, and that Hm-MyD88 and Hm-SARM (sterile α and Toll/IL-1R resistance motif-containing protein) are involved in the processes of immune stress and CNS repair (68). Notably, the regeneration of severed nerve cords appears to proceed more rapidly upon pathogen stimulation, which may be associated with the Hm-TLR1-induced upregulation of pro-inflammatory cytokine p43 (66, 67). Overall, these results indicate that annelid TLRs play vital roles in neurogenesis and neuroimmunity.

### Mollusca

Until now, several TLRs have been identified in molluscan species, including *Crassostrea gigas, Biomphalaria glabrata, Chlamys farreri*, and *Cyclina sinensis*. There are 56 TLR-encoding genes in *B. glabrata* genome, 27 of which encode complete TLRs, including 25 V-type TLRs and 2 P-type TLRs (69). A novel snail TLR (Bg-TLR) has been identified in *B. glabrata*, which is overexpressed after stimulation with the parasitic platyhelminth *Schistosoma mansoni*. A knockdown of *Bg-TLR* results in increased susceptibility of *B. glabrata* to parasites, indicating that Bg-TLR may play vital roles in the immune response of *B. glabrata* following an infection (70). In addition, it has been reported that Bg-TLR is dramatically overexpressed in hemocytes upon PAMP stimulation, and its knockdown impairs the hemocyte phagocytic activity (70, 71). A recent phylogenetic analysis revealed that a V-type TLR (TLR2) of *B. glabrata* does not cluster together with other molluscan V-type TLRs because of its LRR N-terminal domain (63).

In total, 83 TLR genes (5 V-type TLRs, 4 P-type TLRs, and 74 V- and P-type variants) and 4 TIR domain-containing proteins have been identified in the Pacific oyster *C. gigas* (72). The NF-κB signaling pathway is activated when five *C. gigas* V-type TLRs are overexpressed in HEK293 cells, especially when co-expressed with *C. gigas* MyD88 (24). In addition, the expression of P-type CgToll-1 is induced during *Vibrio anguillarum* infection (73). Similarly, treatment of *C. gigas* hemocytes with heat-inactivated *Vibrio parahaemolyticus* also upregulates the expression of five V-type TLRs (24, 73). To date, two V-type TLRs (TLR4 and TLR13) and a MyD88 gene have been identified in the clam *C. sinensis* (74). The presence of a pathogen-responsive TLR13-MyD88-NF-κB pathway in the hemocytes of *C. sinensis* has been demonstrated, and knockdown of TLR13 results in the reduced expression of all other adaptors involved in this signaling network (75). Furthermore, infection of *C. sinensis* hemocytes with *Micrococcus luteus* or *V. anguillarum* induces the expression of TLR13, MyD88, and NF-κB, whereas this transcriptional response is abolished upon knockdown of TLR13 (74, 75). These results indicate that MyD88-dependent signaling pathway is involved in the activation of downstream immune reactions in *C. sinensis*, especially the antibacterial response.

A single P-type TLR and four LRR domain-containing proteins have been identified in the scallop *C. farreri* (76–78). The interaction between the TIR domain of *C. farreri* P-type TLR (CfTLR-TIR) and *C. farreri* MyD88 has been verified, indicating that MyD88 may participate in CfTLR-induced signaling (79). The expressions of CfTLR, CfMyD88, CfTRAF6, CfNF-κB, and CfIκB were upregulated upon stimulation of *C. farreri* with LPS, and *C. farreri* became more susceptible to *Listonella anguillara* infection when *CfTLR* was silenced (79). The CfTLR network also plays a role in hemocytes, as the expressions of pathway components, as well as that of four LRR-domain containing proteins, are induced upon exposure to several pathogens (77, 78). Furthermore, a previous study demonstrated that the NF-κB signaling is activated when the ectodomain of CfTLR is stimulated with various TLR ligands in HEK293 cells, suggesting that CfTLR can directly sense PAMPs (80). Collectively, these results indicate that the TLR signaling network of *C. farreri* plays pivotal roles in the immune defense against pathogens and PAMPs, through a direct-sensing manner.

In summary, these studies revealed that molluscan TLRs are capable of activating MyD88-dependent NF-κB signaling upon stimulation by pathogens. Furthermore, molluscan TLRs likely play an important role in molluscan development, as demonstrated by the upregulation of three *C. gigas* TLRs during early embryonic development (72). However, there have been relatively few studies concerning the developmental roles of molluscan TLRs and thus, further studies are essential to elucidate these issues.

### Arthropoda

#### Merostomata

Toll-like receptors in Merostomata species have been, to date, investigated by relatively few studies. A TLR gene (*tToll*), with the length and architecture comparable with that of Toll-1 of *Drosophila*, has been identified in the horseshoe crab *Tachypleus tridentatus* (81). The tToll-1 protein contains LRRs domain with a length of 22–25 residues; however, these LRRs do not appear to play a role in PAMP binding but instead, tend to bind to molecules resembling *Drosophila Spätzle* (81, 82). Coagulin reportedly enhances the dimerization of tToll and induces the activation of intracellular cascades (83). In *Carcinoscorpius rotundicauda*, a TLR signaling adaptor SARM has been identified, the expression of which is rapidly upregulated 3 h after *P. aeruginosa* infection and then strongly inhibited 6 h after the infection. Furthermore, CrSARM reportedly inhibits activation of the TLR pathway by interacting with TRIF in HEK293 cells, indicating that the function of this adaptor is conserved from arthropods to mammals (84).

#### Insecta

With more than a million species, Insecta is by far the largest group of hexapod invertebrates within Arthropoda. Upon infection, insects can rapidly mount an antimicrobial response involving cells, components, and processes, such as hemocytes, antimicrobial cytokines, and melanization, respectively (85–88). The insect antimicrobial response appears to be similar to the mammalian innate immune response (89). To date, studies of the model species *D. melanogaster* have provided a foundation for the understanding of fundamental mechanisms of the immune response in insects. It has been reported that the innate immune response of *D. melanogaster* relies to a great extent on a wide variety of antimicrobial peptides that function synergistically to counter pathogen infection (85–88). In particular, studies have indicated that the Toll pathways are involved in the induction of expression of antimicrobial peptides in *D. melanogaster* (90, 91).

In 1985, Toll-1 (the first TLR ever identified) was discovered in *D. melanogaster* embryos, based on its role in the specification of the embryonic dorsal ventral polarity (3). Thereafter, genes for other Toll family members (Toll-2–9) were detected in the genome of *D. melanogaster* (92, 93), and their dual function in immune response and embryogenesis has been gradually confirmed (94). Imler and Hoffmann have reported that, with the exception of Toll-9, all Toll proteins possess 1–4 additional cysteine-rich motifs (95).

In *D. melanogaster*, the Toll-dependent pathway mediates activation of NF-κB in response to Gram-positive (G^+^) bacteria and fungi, whereas the parallel immune deficiency (IMD) pathway responds to G^–^ pathogens (90, 94). In contrast with mammalian TLRs, *D. melanogaster* Toll proteins do not directly bind to any PAMPs and their activation requires the cleavage of the accessory protein Spätzle. Gram-negative binding protein (GNBPs) and persephone or peptidoglycan recognition proteins (PGRPs) are responsible for recognizing G^+^ bacteria or fungi, respectively (96, 97). G^+^ bacteria-derived Lys-type peptidoglycan can be recognized by the upstream receptors PGRP-SA and PGRP-SD, as well as by GNBP-1 (98–100). PGRPs and GNBPs can activate proteolytic cascades, which finally cleave Spätzle. The cleaved form of Spätzle directly binds to Toll and activates the Toll-to-NF-κB signaling pathways, leading to enhanced transcription of multiple target genes of *D. melanogaster*. Persephone is a protease, hence it can directly cleave Spätzle and activate the Toll signaling. Hu and colleagues demonstrated that the Toll-Spätzle complex exists in a 2:2 form, with two sites of interaction between the ectodomain chains (101). In addition to initiating the innate immune responses induced by fungi and G^+^ bacteria, the *Drosophila* TLR-to-NF-κB pathway also plays an essential role in the establishment of the early embryonic dorsal–ventral polarity (93, 102–104). Moreover, several *Drosophila* TLRs have important functions related to the maintenance of tissue health by inducing NF-κB-dependent apoptosis of unfit or mutant cells (105).

#### Crustacea

To date, several TLRs have been identified in crustacean species, including crabs, shrimps, and copepods. Among these, TLRs in shrimps, including *Litopenaeus vannamei, Procambarus clarkii, Penaeus monodon, Fenneropenaeus chinensis, Macrobrachium rosenbergii*, and *Marsupenaeus japonicus*, are the most extensively studied (106–112). Three TLRs (Toll-1–3) have been identified in *M. japonicus*, and can be activated by G^+^ and G^−^ bacterium infection. Unlike the requirement for Spätzle of *Drosophila* Toll, shrimp Toll proteins can bind directly to PAMPs from G^+^ and G^−^ bacteria, which leads to the upregulation of the expression of various antibacterial peptides to clear the invasive bacteria (112). Consistent with other species, NF-κB is a typical downstream transcriptional factor in the Toll signaling pathway of shrimps (113–115). Yao and colleagues revealed that genetic polymorphisms of TLRs are linked to the immune response to pathogen infections in *L. vannamei* (116). Deepika and colleagues suggested that the TLR-TRAF6-mediated signaling pathway in *P. monodon* functions as a pivotal component in the defense against viruses (117). Recently, a Toll-9 receptor, PmToll9, has been identified in *P. monodon*, and it has been demonstrated that PmToll9 can induce the downstream Toll pathway cascades, leading to the activation of NF-κB (118). However, the mechanisms underlying the role of PmToll9 in defending the host against pathogens and mediating the innate immunity require further exploration.

Studies of TLRs in crabs have mostly focused on mud crabs, including *Scylla paramamosain, Scylla serrata*, and the Chinese mitten crab *Eriocheir sinensis* (119–123). Challenge of *S. paramamosain* with the bacterium *Vibrio harveyi* enhances the expression of both, SpMyD88 and SpToll, whereas that with *Staphylococcus aureus* only induces upregulation of SpMyD88 levels. Interestingly, SpMyD88 can associate with SpToll (119). In addition, a single-nucleotide polymorphism mutation (c.1372A > G) in SpToll may enhance the resistance of crabs to pathogens (124). Apart from SpMyD88 and SpToll, another characterized gene product implicated in TLR cascades is Sp-TRAF6, which plays a critical role in host defenses by regulating ALF genes (125). In addition, it has been reported that TLR signaling pathway-related genes in *Caligus rogercresseyi* are highly expressed at the chalimus and adult stages, promoting a more developed immune response (126).

### Echinodermata

Echinoderms are the most evolutionarily advanced invertebrates and share the evolutionary history with chordates. TLRs reportedly play a pivotal role in the immunity of metazoans, including echinoderms, such as sea urchins and sea cucumbers (127). Analysis of the entire genome of sea urchins, especially that of the purple sea urchin *S. purpuratus*, has been attempted in several studies. The innate receptor repertoire of *S. purpuratus* is vastly expanded, and this has not been previously observed in any other species. Overall, 222 TLR-like genes have been detected in the *S. purpuratus* genome (8 P-type TLRs and 214 V-type TLRs) that can be classified into seven major groups by molecular phylogenetic tree analysis (127, 128). Buckley and colleagues reported that even though only 68 TLR-like gene sequences were found in *Lytechinus variegatus*, majority of the TLR subfamilies and homologous sequences identified in *S. purpuratus* were also present in *L. variegatus* (127). In addition, analysis of the *S. purpuratus* genome has led to the identification of homologs of several TLR signaling-related proteins, including MyD88, SARM, THF-α, and NF-κB (127–129). Roberta and colleagues identified a novel mRNA sequence in the immune cells of *Paracentrotus lividus*, referred to as Pl-TLR, which encodes a TLR protein. The mRNA levels of Pl-TLR were apparently elevated by stimulation with poly (I:C), in a time-dependent manner. By contrast, LPS treatment did not significantly alter the Pl-TLR mRNA levels (130), which suggested that Pl-TLR might not be associated with the immune response induced by bacteria. In echinoderms, it appears that members of the TLR3 family are specifically responsive to viral dsRNA associated with viral infection, whereas TLR4 receptor subfamily members are particularly responsive to LPS stimulation (131).

In echinoderms, studies have also focused on the TLRs of *Apostichopus japonicas*. It has been reported that the structures of AjMyD88 and AjTRAF6 and those of MyD88 and TRAF6 proteins of other species are highly conserved, and that the expression of genes encoding these two proteins is significantly upregulated following stimulation with *Vibrio splendidus* (132). Wang and colleagues suggested that Aj-rel and Aj-p105 (two evolutionarily conserved NF-κB homologs) share many characteristics with their vertebrate orthologs, and that stimulation with LPS induces Aj-p105 degradation, and nuclear translocation of Aj-rel and Aj-p50 (133). A recent study of *A. japonicus* has revealed that *V. splendidus* and LPS treatment induce marked increase in the expression of AjHMGB3 and AjHMGB3 through the activation of a TLR cascade (134). Similarly, AjTLR3 and AjToll, which harbor LRR, TM, and TIR domains, have been shown to be involved in immune defense responses against various bacteria and viruses (135).

## TLRs in Amphioxus

Evolutionarily placed at the invertebrate–vertebrate transition point, amphioxus, a typical cephalochordate, represents an important organism for research into understanding the evolution of the TLR-associated immune system (136). Big Bang expansion of the V-type TLRs in amphioxus has been reported. The amphioxus genome encodes an extraordinarily complex TLR system, including at least 48 TLRs and more than 40 TIR adaptors (137). The draft genome of the amphioxus encodes at least 36 V-type TLRs and 12 P-type TLRs, suggesting that the amphioxus V-type TLR lineage has also greatly expanded and the P-type TLR structure may have been only lost in vertebrates (137). Subsequently, an immune-associated TLR1 that participates in the defense against certain pathogens has been identified in *Branchiostoma belcheri tsingtauense* (bbt) (138). In general, most TLRs depend on a family of adaptor proteins containing the TIR domain for signal transduction (139). Up to date, three TIR adaptors of amphioxus have been well studied, namely, MyD88, TICAM, and SARM. The middle and death domains of bbtMyD88 have been shown to be associated with NF-κB activation in response to bacteria and their cell wall components, whereas bbtTICAM activates NF-κB in a MyD88-independent manner by interacting with RIP *via* its RHIM motif (136, 138, 140). Further, bbtTICAM shares amino acid similarity with mammalian TICAM1 (also known as TRIF) and TICAM2, hence, perhaps it is the ancestor and ortholog of these proteins. Surprisingly, unlike its mammalian counterparts, bbtTICAM does not induce the production of IFN1, indicating that the antiviral IFN system has not evolved prior to vertebrates (136, 138, 140). Amphioxus SARM plays an inhibitory role in both TICAM- and MyD88-dependent pathways, by interacting with TRAF6, MyD88, and TICAM (140, 141). Furthermore, Peng and colleagues found that bbtTIRC inhibits bbtMyD88-mediated signaling by interacting with bbtMyD88 and suppressing bbtTRAF6 polyubiquitination, whereas bbtTIRA inhibits bbtTICAM-mediated activation of NF-κB through interacting with both bbtRIP1b and bbtTICAM (142). Since genes encoding bbtTIRA and bbtTIRC are NF-κB targets, these two proteins are likely to constitute an effective feedback regulation mechanism of amphioxus NF-κB signaling. In addition, according to several studies, ubiquitination plays an essential role in regulating NF-κB activation in amphioxus (143, 144). Collectively, these findings provide reference for studying the complexity of the amphioxus innate immunity and indicate new perspectives for the related studies of vertebrates.

## TLRs in Non-Mammalian Vertebrates

Non-mammalian vertebrates include organisms from the classes Cyclostomata, Chondrichthyes, Osteichthyes, Amphibia, Reptilia, and Aves. To date, at least 28 functional TLRs have been identified in various species from these classes. They can be divided into six major subfamilies, namely, the TLR1, TLR3, TLR4, TLR5, TLR7, and TLR11 subfamilies. The large TLR1 subfamily, consisting of TLR1, 2, 6, 10, 14, 15, 16, 18, 25, 27, and 28, mainly recognizes lipoproteins, whereas the TLR3, 4, and 5 subfamilies recognize dsRNA, LPS (although not in fish and amphibians), and bacterial flagellin, respectively. The TLR7 subfamily, which includes TLR7, 8, and 9, plays a role in the recognition of nucleic acid motifs. Members of the sixth major subfamily, the TLR11 subfamily, containing TLR11, 12, 13, 19–23, and 26, have multiple functions, which range from sensing proteins to nucleic acid motifs.

### Cyclostomata

Cyclostomata, the lowest class of vertebrates, consists of two families of surviving jawless fish, the lampreys and hagfish (145). Two TLRs (laTLR14a and laTLR14b) have been identified in the Japanese lamprey (*Lampreta japonica)* by polymerase chain reaction-based cloning (146). Interestingly, TLR14 is a member of the TLR1 subfamily, and the encoding gene which is present in the genomes of teleosts and amphibians (21, 146), suggesting that the current vertebrate TLR subsets emerged before the mammalian ancestor diverged from the jawless fish ancestor (21). Full-length sequences of both laTLR14a and laTLR14b contain eight LRRs, a transmembrane region, and a cytoplasmic TIR domain, and display high similarity. Their TIR domains are also similar to those found in the human TLR1 subfamily, and share 56% similarity to that of pufferfish TLR14 (146). The expression of *latlr14a* is restricted to the gill, while the expression of *latlr14b* is observed in the skin, gill, heart, liver, gut, and leukocytes (146). Subcellular localization analysis indicates that laTLR14a and 14b are largely localized in the endoplasmic reticulum, with only a small fraction found in other organelles. The NF-κB and INF-β gene promoters are activated in a human MyD88-dependent pattern in HEK293 cells upon artificial laTLR14b dimerization (146). However, whether this type of innate immune signaling occurs in the Japanese lamprey *in vivo*, and the identities of PAMPs necessary for activation and the adaptors involved, await further elucidation.

Subsequently, 16 TLR-encoding genes and four genes similar to one encoding MyD88, TICAM, or SARM were identified by Kasamatsu and colleagues (147) by surveying the amino acid sequences of TIR-containing proteins in the sea lamprey (*Petromyzon marinus*) genome database and NCBI trace archive. The repertoire of the 16 predicted lamprey TLRs has been determined. Phylogenetic analysis indicates that this group comprises both fish-type TLRs and mammalian-type TLRs. Three types of sea lamprey TLRs belong to the TLR1 subfamily, namely, TLR14 (pmTLR14a-c), TLR24 (pmTLR2a-d), and an ortholog of the jawed vertebrate TLR14 (TLR14d). Similarly, genes encoding one TLR3, one TLR5, two TLR7/8, and three TLR21 have also been identified in the sea lamprey genome. Additional two TIR domain-containing molecules, pmTICAM1a and pmTICAM1b, have also been identified, and the latter was shown to be an ortholog of the jawed vertebrate TRIF. However, orthologs of TLR4, TLR9, and TLR15, and TLR4-related genes appear to be absent in the sea lamprey, indicating the lack of a traditional LPS-recognition TLR4 system in jawless vertebrates. Furthermore, none of the genes required for IFN1 induction, including ones encoding IRF-1, IRF-3, IRF-7, and IFN1, have been identified in sea lamprey. Hence, the IFN induction pathway may have originated in a common ancestor of jawed vertebrates.

### Chondrichthyes

Jawed cartilaginous fish (Chondrichthyes) represent an important group of animals for immune-related research. They are considered to be the first species to have evolved adaptive immune responses. Further, the presence of the innate immune system at this evolutionary turning point is intriguing. However, relatively few studies have focused on the TLRs of cartilaginous fish and, accordingly, there is a need to fill the gaps in the current knowledge about this group. TLR2, TLR3, TLR6, and TLR9 have been identified in the gray bamboo shark *Chiloscyllium griseum* based on a survey of transcriptome data (148, 149). TLR2 of *C. griseum* is closely related to homologs in *Sus scrofa* and *Gallus gallus*, whereas TLR3 is closely related to homologs in *Rattus norvegicus* and *Canis lupus familiaris*. TLR6 shows the highest similarity with homologs in *Bos tarus* and *Felis catus*, and TLR9 with homologs in *Andrias davidianus*. Further protein modeling analysis indicated that *C. griseum* TLRs could bind poly (I:C), similarly to their mammalian homologs. Furthermore, sequence for adaptors of the TLR3 signaling pathway, including TRAF3, TBK1, IRF3, and IRF7, have also been found in the *C. griseum* transcriptome, indicating evolution of the IFN1 pathway. In addition, two genes encoding TIR domain-containing molecules (TRIF and TRAM) and one MAL have been identified in the *Callorhinchus milii* genome (150). As shown in mammals, TRIF is involved in both TLR3 and TLR4 signaling pathways, TRAM is essential for TRIF-dependent TLR4 signaling, and MAL is involved in MyD88-dependent TLR4 signaling. Hence, a relatively mature TLR3 and TLR4 network may have evolved in Chondrichthyes, and the encoding genes (TRIF, TRAM, MAL, genes for IFN1 production) may have co-evolved with TLR3 antivirus signaling and TLR4 signaling. However, the exact functions of these shark TLRs and their PAMP repertoire, as well as the induced signaling pathways require further experimental verification.

### Osteichthyes

Osteichthyes, also referred to as teleost fish, comprise a remarkably diverse group of more than 23,500 species (151). To date, 21 TLRs (TLR1–5, 5S, TLR7–9, TLR13, 14, TLR18–23, and TLR25–28) have been identified in many different teleost fish species, including both, orthologs of mammalian TLRs and "teleost-specific" TLRs (22, 152–154). Among these, the structural and functional properties of TLR1–3, 5, and 7–9 are similar to those of their mammalian counterparts, whereas teleost TLR4 appears to be structurally conserved, but does not recognize LPS, unlike in mammals. To date, no TLR6 or TLR10 homologs have been identified in teleost fish. "Teleost-specific" TLRs include TLR5S, 18–20, 23, and TLR25–28; however, although these are designated as "specific," they nevertheless show high structural similarity to the mammalian TLR system (155).

As mentioned above, vertebrate TLRs can be divided into six major subfamilies (156). The teleost TLR1 subfamily members include TLR1, TLR2, TLR14, TLR18, TLR25, TLR27, and TLR28. In mammals, TLR1 is involved in the recognition of triacylated lipoproteins and mycobacterial products by binding to TLR2 to form a heterodimer (157). Teleost TLR1 and TLR2 have been characterized in Tetraodon (*Tetraodon nigroviridis*) (157), pufferfish (*Takifugu rubripes*) (158), zebrafish (*Danio rerio*) (159, 160), Japanese flounder (*Paralichthys olivaceus*) (161), channel catfish (*Ictalurus punctatus*) (152, 162), rainbow trout (*Oncorhynchus mykiss*) (163, 164), orange-spotted grouper (*Epinephelus coioides*) (165), large yellow croaker (*Pseudosciaena crocea*) (166–168), rohu (*Labeo rohita*) (169), common carp (*Cyprinus carpio*) (170), and grass carp (*Ctenopharyngodon idella*) (171). TLR2 signaling plays an important role in the activation of intestinal immune system in mammals (172). According to a recent study, the TLR2 signaling pathway may be involved in the recognition of probiotic *Psychrobacter* sp. and mucosal immune activation in orange-spotted grouper. TLR14 shares some features with TLR1, TLR6, and TLR10, and has been found in pufferfish and Japanese flounder (21, 173, 174). Teleost TLR14 is reportedly more closely associated with the immune response against G^−^ bacterial infection than against G^+^ bacterial and viral infections (173). TLR18 is an extensively expressed fish-specific TLR that plays a key role in the innate immune responses in teleosts (175). It has been identified in many species, including channel catfish (154), zebrafish (160), grass carp (171), yellow catfish (*Pelteobagrus fulvidraco*) (175), Atlantic salmon (*Salmo salar*) (176), Japanese sea bass (*Lateolabrax japonicus*) (177), and Atlantic cod (*Gadus morhua*) (178). It has been reported that TLR18 in zebrafish and channel catfish are homologs of human TLR1 and may correspond to the TLR14 of other fish (160, 179). Recently, two new TLR types (TLR25 and TLR26) were identified in catfish, the TIR domains of which include numerous conserved regions previously reported in TLRs (152). TLR25 belongs to the TLR1 subfamily, and in addition to catfish, has also been found in Nile tilapia (*Oreochromis niloticus*), fat head minnow (*Pimephales promelas*), ayu (*Plecoglossus altivelis*), and medaka (*Oryzias latipes*). TLR27 was first identified in the coelacanth (*Latimeria chalumnae*) (153). Wang and colleagues (180) recently reported its expression in spotted gar (*Lepisosteus oculatus*), which indicates that TLR27 is highly evolutionarily conserved. More recently, a novel TLR1 family member (designated TLR28) has been discovered in the miiuy croaker (22). Analysis of its characteristics revealed a high homology with TLR2. The protein is highly expressed in the liver of miiuy croaker, and after stimulation with *V. anguillarum, S. aureus*, LPS, and poly (I:C), TLR28 expression is significantly upregulated, indicating the potential role of TLR28 in immune response.

TLR3 is a well-characterized innate immune receptor that senses dsRNA, endogenous cellular mRNA, and sequence-independent small interfering RNAs (181, 182). Like mammalian TLR3, fish TLR3 plays a crucial role in the innate immune responses (183). The TLR3-mediated activation of immune responses in mammals depends on viral dsRNA intermediates (184). Upon binding to TLR3, dsRNA, or its synthetic analog poly (I:C) leads to the induction of IFN1 and inflammatory cytokine production in fish cells (115). In a recent study, Jung and colleagues (185) show that in rock bream (*Oplegnathus fasciatus*), poly (I:C) exerts a biological effect by interacting with TLR3. Presently, TLR3 has been identified in various fish species: zebrafish (186), rainbow trout (187), common carp (188), rare minnow (*Gobiocypris rarus*) (189), grass carp (190), large yellow croaker (191), Japanese flounder (192), and sea perch (*L. japonicus*) (183).

In mammals, members of the TLR4 family are mainly responsible for LPS recognition and are probably the best characterized PRRs (193). However, LPS recognition and sensitivity in fish are fundamentally different from those in mammals. Fish are often resistant to the exotoxin LPS (194). In mammals, a key step in LPS recognition is the transportation of LPS aggregates to the cell surface where they form a ternary complex with CD14, which facilitates the transfer of monomeric LPS to TLR4 and MD2 (195). Sepulcre and colleagues (196) have reported that the sequenced fish genomes lack the genes of co-stimulatory molecules MD2 and CD14. Additionally, the majority of teleost species have lost TLR4 after separation from the mammalian lineage. Whole-genome sequencing of pufferfishes (both *T. rubripes* and *T. nigroviridis*) and stickleback (*Gasterosteus aculeatus*) failed to detect TLR4-encoding sequences (158, 197). However, a TLR4 sequence was identified in Chinese rare minnow (*G. rarus*) and two TLR4 genes were detected in the zebrafish genome (TLR4a/b). Recently, Sepulcre and colleagues (196) reported that LPS signals through a TLR4- and MyD88-independent pathway in fish, and that the zebrafish MyD88-dependent signaling pathway is negatively regulated by TLR4. This may partially explain why fish are resistant to endotoxin and supports the speculation that the TLR4–LPS signaling network appeared after the divergence of fish and tetrapods. Huang and colleagues have identified four *tlr4* genes in grass carp, which differ with respect to genomic structures and protein domains (198). The expression of these four *tlr4* genes is detected 12 h post-fertilization, and is significantly upregulated in the muscle and liver of adult grass carp after infection with grass carp reovirus (GCRV), indicating that these TLR4 homologs may play immune functions during GCRV infection. This implies that ligand specificities of TLR4 proteins of grass carp are different from those of mammalian TLR4, and provides important clues for the evolutionary scenario of TLR4.

Only one member of the TLR5 subfamily, TLR5, is found in mammalian species (199). Tsujita and colleagues reported for the first time that the TLR5 protein is present in rainbow trout in both membrane-bound form (TLR5M) and non-transmembrane soluble form (TLR5S) (200). TLR5M is similar to the mammalian TLR5, containing the typical LRRs, transmembrane region, and conserved intracellular TIR domain. TLR5S lacks the transmembrane region and the intracellular TIR domain (201). In recent years, TLR5M and TLR5S, which are present in many tissues (158, 200, 201), have been identified in pufferfish (158), orange-spotted grouper (201), Atlantic salmon (202), Japanese flounder (203, 204), catfish (205), Tibetan schizothoracine fish (197, 206), and turbot (207). Previous studies on Atlantic salmon, Japanese flounder, pufferfish, and catfish revealed that TLR5M contains 9–12 LRR domains, and that the TIR domain contains 139–151 amino acids. TLR5M is ubiquitously expressed in teleosts, with relatively high expression levels in the head kidney, spleen, liver, and brain tissues, whereas TLR5S has been detected mainly in the liver (201). Zebrafish TLR5M primarily binds to the D1 domain of flagellin and forms a simple heterodimer. Two heterodimers form a stable complex *via* tail-to-tail binding that is stabilized by quaternary contacts of the FliC D1 domain with the convex surface of the opposing TLR5 and then induces MyD88-dependent signaling (197, 208). Rainbow trout TLR5S is also involved in the activation of NF-κB pathway in response to flagellin (209). Hence, in teleost fish, TLR5S may recognize a variety of bacterial flagellins to augment NF-κB activation (200, 209). Recognition of PAMPs or DAMPs by TLR5 leads to activation of MyD88-dependent TLR signaling pathway during cold and heat shock, as has been characterized in the Indian major carp (210).

The TLR7 subfamily contains TLR7, TLR8, and TLR9, which are structurally similar to the mammalian homologs and have 13–15 LRR domains (160, 194, 211–214). In recent years, TLR7–9 have been identified in channel catfish (152), Atlantic cod (178), Tibetan schizothoracine fish (206), common carp (211), zebrafish (160), rainbow trout (215), grass carp (216, 217), Atlantic salmon (218, 219), yellow catfish (220), turbot (221), flounder (222), and sea bream (223). Kileng and colleagues found that the imiquimod derivative S-27609 induces the upregulation of IFN-α1/α2 and IFN-γ expression in the liver and head kidney tissues in Atlantic salmon through a TLR7-like receptor, providing indirect evidence for the presence of TLR7 signaling pathways in fish (214). After 8-week infection with the pathogen *Mycobacterium marinum*, the expression of TLR7 in the blood and lymphoid tissues of zebrafish is reduced, whereas the expression of TLR8 is elevated (160). Poly (I:C) stimulation of large yellow croaker results in elevated expression of TLR7 and TLR8 in the head kidney and spleen tissues (213). These differences in expression patterns may be related to the type of fish and different mechanisms of regulation *in vivo* and *in vitro*. Studies on the TLR9 genes in common carp (211), large yellow croaker (212), and gilthead sea bream (223) have indicated that TLR9 is mainly expressed in the kidney and spleen tissues, and that its expression increases following *V. parahaemolyticus* infection. Distinct expression patterns of TLR8 and TLR9 have been observed in the mucosal tissues (the intestine, gill, and skin) of turbot after infection with *V. anguillarum* and *Streptococcus iniae* (221). In addition, stimulation with G^−^ pathogens, IFN-γ, and CpG induces TLR9 gene expression in teleost fish (21, 211). Given that the genes for the fish TLR7 subfamily respond to both viral stimuli and bacterial infections, it is possible that their functional differentiation may not be as precise as that of mammalian TLR7 subfamily members.

Numerous "fish-specific" TLRs have been isolated, including TLR13 (224, 225), TLR19 (23, 226), TLR20 (197, 203, 227), TLR21 (158, 197, 203, 228), TLR22 (55, 229–231), TLR23 (158), and TLR26 (152), in such fish as miiuy croaker, punctate-tailed fish, zebrafish, pufferfish, large yellow croaker, Atlantic salmon, Asian sea bass, orange-spotted grouper, grass carp, and barbel chub. Wang and colleagues were the first to report that in Perciformes (particularly Sciaenidae), the teleost TLR13 is highly expressed in such immune defense-related tissues as the spleen, liver, and kidney (224). Subsequently, TLR13 was identified in orange-spotted grouper, with relatively high expression levels in the brain and immune-related tissues, and was also found to be significantly upregulated in grouper spleen cells, indicating that TLR13 may be involved in the recognition of bacterial RNA (225). Teleost-specific TLR19 is localized in the endosomes, recognizes dsRNA analogs, promotes IFN and NF-κB expression, and protects the cells from GCRV infection (23). When challenged with poly (I:C) or *Aeromonas hydrophila*, TLR22 is upregulated in a variety of common carp tissues (231), indicating an important role of TLR22 in systemic as well as mucosal defense after viral or bacterial infection. In a recent study, TLR22 was shown to be an intracellular receptor localized in the endosomes, which functions as an inflammation equalizer *via* selective activation of the MAPK pathway and suppression of NF-κB in fish (232). Transcript levels of *TLR22* are increased in response to bacterial-borne PAMPs and extracellular dsRNA in the euryhaline teleost Asian sea bass, with the highest expression observed in the kidney and liver (229). However, the mechanisms underlying TLR11 subfamily function in fish require further investigation.

### Amphibian

To date, at least 20 TLRs (TLR1, 2.1–2.2, 3–5, 6.1–6.2, 7, 8.1–8.2, 9, 12, 13, 14.1–14.4, 21, and 22) have been identified in amphibians (146, 153). In addition, several types of soluble LRR-only TLRs have been reported (21). TLR2, TLR6, and TLR8 may be duplicated in amphibians. The TLR14 subfamily appears to have been lost in amniotes but is expanded in amphibians. Among the TLRs that are present in mammals but absent in teleosts, orthologs of mammalian TLR6 and TLR12 have been identified, whereas those of mammalian TLR10 and TLR11 have not been detected. Additionally, amphibians harbor a putative soluble short form of TLR5 (TLRS5) (21). The presence of *tlr4* in *Xenopus* genome has been confirmed, but not CD14 or MD2 (essential for TLR4-mediated recognition of LPS). Therefore, *Xenopus* TLR4 might not be responsible for the bacterial exotoxin-initiated signaling pathway. Furthermore, Ishii and colleagues reported that the size and number of LRRs of all *X. tropicalis* TLRs are similar to those of the human TLR counterparts. They also confirmed that genes of these TLRs are ubiquitously expressed in both tadpole and adult frog (146). The role of TLR signaling pathway in dorsoventral patterning (233), and the involvement of MyD88 in the Spemann organizer formation have been reported in *Xenopus laevis*, suggesting that the TLR signaling is also essential for amphibian development (234). Recently, an adaptor molecule MyD88 of *Rana dybowskii* was cloned and analyzed, and shown to contain a death domain and a TIR domain (235). The structure of MyD88 in *R. dybowskii* is conserved, and the protein probably participates in the antiviral and antibacterial immunity of *R. dybowskii*.

TLR7 (CgsTLR7), containing 19 LRRs and a TIR domain, has recently been identified in Chinese giant salamander *A. davidianus* (236), and is found to have two transmembrane domains similar to those identified in frogs (146, 236). Moreover, the expression of CgsTLR7 is upregulated in the liver, kidney, and spleen in response to a giant salamander iridovirus infection, indicating that CgsTLR7 plays vital roles in innate immunity (236).

### Reptilia

As the only poikilothermic amniotes, reptiles have a unique physiology and occupy a central position in vertebrate evolution. However, the structure, function, and ligand specificity of TLRs in reptiles have not been characterized (237). Searches for reptilian TLR sequences have identified these in only one species, the green anole lizard *Anolis carolinensis*, which have been annotated as molecules resembling mammalian TLR2, 3, 4, 5, 6, 7, and 13. Recently, Voogdt and colleagues (170) reported the cloning, characterization, and functional analysis of TLR5 from *A. carolinensis*, and found that the receptor (acTLR5) displays a typical TLR protein structure with 22 extracellular LRRs flanked by N- and C-terminal LRR domains, an intracellular TIR domain, and a transmembrane region. Phylogenetically, acTLR5 is most distant from the TLR5 of fish and closest to the avian TLR5. Stimulation experiments of acTLR5 with PAMPs revealed a unique responsiveness toward bacterial flagellin.

### Aves

Although diverged about 300 million years ago, the immune responses of avian are broadly similar to those of mammals (238, 239). Most of the knowledge of avian immunology was generated by studies of the junglefowl *G. gallus*, the ancestor of domestic chicken (240). To date, much has been learned about avian TLRs with regards to the recognized ligands.

Avian genome analysis has more recently been extended from *G. gallus* to the Zebra finch *Taeniopygia guttata*, a species that diverged from the *G. gallus* lineage approximately 100 million years ago, to obtain a more comprehensive understanding of avian TLR repertoire (238). Several studies have confirmed the presence of 10 avian Toll-like receptors (TLR1La, TLR1Lb, TLR2a, TLR2b, TLR3, TLR4, TLR5, TLR7, TLR15, and TLR21); structurally, 6 of them (TLR2a, TLR2b, TLR3, TLR4, TLR5, and TLR7) are clearly orthologs of mammalian TLRs (238, 241–244). Gene duplication resulted in the occurrence of TLR1La and TLR1Lb, and TLR2a and TLR2b in *T. guttata*. Avian TLR21 is an ortholog of TLR21 identified in teleosts and amphibians, whereas avian TLR15, which belongs to the TLR1 subfamily, appears to be unique to avian species. The expression of TLR4 and MD2 was demonstrated in chicken, where these proteins are involved in LPS-stimulated activation of NF-κB but not the production of IFN1 (245). However, stimulation of chicken leukocytes with poly (I:C) and the subsequent upregulation of IFN1 suggest the existence of TRIF signaling pathway (238). Hence, the chicken immune system may respond to LPS in a MyD88-dependent TRIF-independent manner. The absence of TICAM2 ortholog in the chicken genome may partially explain why, unlike in mammals, TRIF does not participate in LPS–TLR4 signaling (238).

A study conducted to characterize TLRs in seven distantly related avian species—*Carpodacus mexicanum* (Fringillidae), *Falco naumanni* (Falconidae), *Accipiter cooperii* (Accipitridae), *Oceanodroma leucorhoa* (Hydrobatidae), *Amazona albifrons* (Psittacidae), *Dromaius novaehollandiae* (Casuariidae), and *Picoides pubescens* (Picidae)—revealed that avian TLRs appear to be characterized by purifying selection, although positive selection patterns have acted on specific amino acid residues. Moreover, many positively selected positions have been mapped to putative ligand-binding regions, indicating that the variations are related to species-specific differences in the recognition of PAMPs (246).

## Evolutionary Scenario for Animalia TLRs and Signaling Pathways

Animalia TLRs can be divided into two basic categories, the P-type and V-type. Most insect TLRs represent the P-type, while all the vertebrate TLRs are V-type (24). The most ancient TLR-like molecules have been discovered in Porifera: instead of the canonical three structural domains, they were TIR domain-containing proteins with N-terminal IL-1R–like Ig domains and LRR-containing proteins with Ig- and EGF-like domains (26–28). Later, canonical TLRs were identified in Cnidaria (34), while the ancestral type still exists until the phylum Annelida (64). The emergence of typical TLRs may be caused by genetic recombination of the ancestral TIR domain-containing type and LRR domain-containing type through their common Ig domains.

A Big Bang expansion of V-type TLRs was reported in many invertebrates including sea urchin and amphioxus *via* specific gene duplication (128, 137), which may be associated with the variation of life cycles, lifetimes, or environments. Those TLRs are characterized by rapid sequence divergence within the LRR domain and conservation within the TIR domain (127). This could be explained by the process of diversifying selection, as the LRR domain is responsible for pathogen recognition and its diversity is most likely caused by the positive selection of pathogens. During the life time of invertebrates, those closely related but diversified variants of TLRs could respond to the enormous and quickly evolving pathogens, and even the change of environments. However, further functional characterization of those TLRs still needs to verify this hypothesis.

Most invertebrate TLRs play dual roles and participate in both, developmental processes and immune responses against pathogens (Table 1). However, the function of vertebrate TLRs is specific to immunity, except for amphibians, where it is involved in dorsoventral patterning and Spemann organizer formation. At least 28 TLRs has been identified in vertebrates and can be divided into six major subfamilies (Table 2). Although some TLRs are not present in mammals, they nevertheless share high structural and functional similarities with mammalian TLRs. Compared with other vertebrates, teleosts and amphibians have the most complex TLR repertoire. Such expansion may be associated with the diversity of aquatic pathogens and the complexity of aquatic environment (22, 152–154).

**Table 1:** Summary of toll-like receptors (TLRs) in invertebrates and amphioxus.

Organism	TLR numbers	Adaptors	Biological roles
**Porifera**			
*Amphimedon queenslandica*	2 (non-canonical)	MyD88	Development
*Suberites domuncula*	1 (non-canonical)	MYD88	Immunity
**Cnidaria**			
*Hydra*	4 (non-canonical)	MyD88	Immunity
*Exaiptasia pallida*	2 (non-canonical)	–	–
*Nematostella vectensis*	1 (canonical)	MyD88	Immunity, development
*Acropora digitifera*	17 (4 canonical)	–	–
*Acropora millepora*	11 (1 canonical)	–	–
*Orbicella faveolata*	5 (1 canonical)	MyD88	Immunity
**Platyhelminthes**			
*Schmidtea mediterranea*	>2 (non-canonical)	MyD88	Regneration
**Nematoda**			
*Caenorhabditis elegans*	1 (canonical)	–	Development, immunity
**Annelida**			
*Helobdella robusta*	16 (8 canonical)	–	–
*Capitella capitata*	105 (23 canonical)	–	–
*Helobdella medicinali*s	>1 (canonical)	MyD88	Neuroimmunity, neurogenesis
**Mollusca**			
*Biomphalaria glabrata*	53 (27 canonical)	-	Immunity
*Crassostrea gigas*	83 (9 canonical)	MyD88	Immunity, development
*Cyclina sinensis*	>2 (canonical)	MyD88	Immunity
*Chlamys farreri*	>5 (1 canonical)	MyD88	Immunity
**Arthropoda**			
**Merostomata**			
*Tachypleus tridentatus*	1(canonical)	MyD88	Immunity
**Insecta**			
*Drosophila melanogaster*	9 (canonical)	MyD88	Immunity, development
**Crustacea**			
*Marsupenaeus japonicus japonicas*	>3 (canonical)	MyD88	Immunity
*Scylla paramamosain*	>1 (canonical)	MyD88	Immunity
**Echinodermata**			
*Strongylocentrotus purpuratus*	222 (canonical)	MyD88	Immunity
*Lytechinus variegatus*	68 (canonical)	–	–
*Apostichopus japonicas*	>2 (canonical)	MyD88	Immunity
**Amphioxus**	>48 (canonical)	MyD88, TRIF	Immunity

*–, represents not characterized*.

**Table 2:** Summary of toll-like receptors (TLRs) in vertebrates.

TLR	Subfamily	Ligand	Cyclostomata	Chondrichthyes	Teleost	Amphibian	Reptilia	Aves	Mammalian
TLR1	TLR1	Lipopeptide			+	+		+	+
TLR2	TLR1	PGN		+	+	+	+	+	+
TLR3	TLR3	dsRNA	+	+	+	+	+	+	+
TLR4	TLR4	LPS^a^			+	+	+	+	+
TLR5	TLR5	Flagellin	+		+	+	+	+	+
TLR6	TLR1	Lipopeptide		+		+	+	+	+
TLR7	TLR7	ssRNA^b^	+		+	+	+	+	+
TLR8	TLR7	ssRNA^c^	+		+	+			+
TLR9	TLR7	CpG		+	+	+			+
TLR10	TLR1	Lipopeptide							+
TLR11	TLR11	Profillin							+
TLR12	TLR11	Profillin				+			+
TLR13	TLR11	Bacterial rRNA			+	+	+		+
TLR14	TLR1	NC	+	+	+	+			
TLR15	TLR1	NC						+	
TLR16	TLR1	Lipopeptide						+	
TLR18	TLR1	NC			+				
TLR19	TLR11	dsRNA			+				
TLR20	TLR11	NC			+				
TLR21	TLR11	CpG			+	+		+	
TLR22	TLR11	dsRNA		+	+	+			
TLR23	TLR11	NC			+				
TLR24	TLR1	NC	+						
TLR25	TLR1	NC			+				
TLR26	TLR11	NC			+				
TLR27	TLR1	NC			+				
TLR28	TLR1	NC			+				

*^a^Only in mammalian and avain does TLR4 recognize LPS (19, 238, 245)*.

*^b^The teleost TLR7 also responds to poly (I:C) stimulation (213)*.

*^c^The teleost TLR8 also responds to poly (I:C) stimulation (213)*.

*NC represents not characterized*.

**Figure 1:**
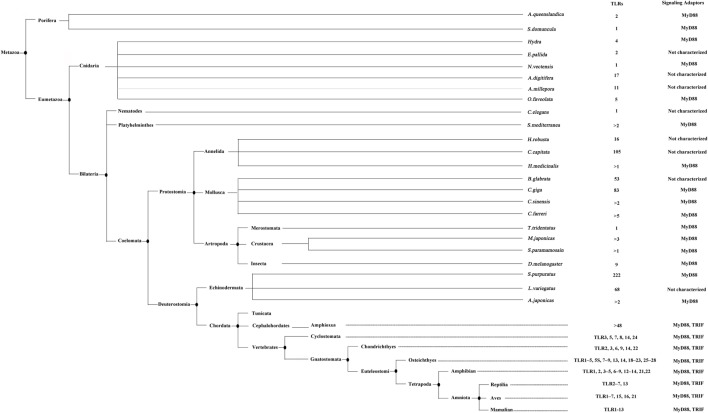
The evolution analysis of toll-like receptors (TLRs) in reported taxa and species. This tree is generated according to the lineages reported by NCBI taxonomy database (http://www.ncbi.nlm.nih.gov/taxonomy). The TLRs identified in different species and the signaling adaptors involved are indicated. This figure is adapted from the publication of MR Coscia et al. (83).

Since the first appearance of TLR in porifera, the animalia has witnessed the development and evolution of TLRs for 1350 million years (Figure 1). Two important questions could be raised regarding the TLR evolutionary scenario. What is the driving force of TLR evolution? What caused the expansion of TLRs in invertebrates and the further contraction in vertebrates? We believe that stress maybe one of the main driving forces in the development of TLRs. The stress conditions, especially challenges to the immune system, such as pathogen, temperature, salinity, and prolonged desiccation, are highly variable and change rapidly. As mentioned above, the pathogen stress may play positive selection roles during TLR evolution. Besides, as the functions of TLRs in invertebrates cover both immunity and development, and the BCR/TCR-mediated adaptive immunity has not evolved yet, the invertebrate TLRs may have been co-opted for use in a strategy that recognize pathogens and exert their bi-functions in a more evolutionary dynamic way than those in vertebrates. Hence, the repertoire of TLRs in invertebrates has expanded to deal with the complex pathogen population and to shoulder the responsibility of development. While in vertebrates, the functions of TLRs are more specialized and restricted to immunity, and also, the specific PAMP recognition mechanism that recognizes pathogens through molecular patterns have been fully adopted. Those limited numbers of PAMPs can be divided into several types and each type can be recognized by one or several types of TLRs. Further, the BCR/TCR-mediated adaptive immune responses have developed in jawed vertebrates and the anti-pathogen immunity no longer relies solely on the innate immune system. Thus, the TLR paradigms in vertebrates have contracted and recognized pathogen in a simpler but more effective manner.

### The Evolutionary Scenario for TLR Signaling Pathways

As mentioned above, the mammalian TLR-mediated pathways can be divided into two categories, MyD88-dependent and TRIF-dependent. Signal transduction in these pathways requires the participation of five TIR domain-containing adaptors, MyD88, MAL, TRIF (TICAM1), TICAM2 (TRAM), and SARM (13, 17). MyD88 mediates a universal pathway for all mammalian TLRs except TLR3. MAL acts as a partner for MyD88 in the TLR4-initiated MyD88-dependent pathway. TRIF is specifically involved in TLR3 signaling and, when coupled to TICAM2, it can also be recruited by TLR4, leading to the production of type I interferon.

It appears that MyD88-mediated signaling pathway is more ancient than the TRIF-mediated pathway. The MyD88 molecule has already appeared in Porifera, the most ancient metazoan phylum showing nucleotide sequences rudiment of TLR, indicating a co-evolution pattern exists between MyD88 and TLRs (26–28). Likewise, the presence of MyD88 and NF-κB in all the phyla of invertebrates, amphioxus, and vertebrates illustrates that this pathway has been highly conserved during evolution. Unlike MyD88, no homolog of TRIF has been identified in non-chordates until its first emergence in amphioxus (bbtTICAM) (136, 140). Previous study has indicated that the mammalian TRIF and TICAM2 were duplicated from a common ancestor (247). Further, bbtTICAM shares amino acid similarity with both mammalian TICAMs. Hence, the mammalian TRIF and TICAM2 may have originated from bbtTICAM through gene duplication and function refinement. TRIF but not TICAM2 has been later identified in all vertebrates. No TICAM2 orthologs have been identified in vertebrates other than mammals and elephant shark (150). TICAM2 plays an essential role in TRIF-mediated TLR4 signaling for LPS recognition, and the binding of LPS to TLR4 is not observed in jawfish and amphibians. Hence, the endotoxin recognition complex and the downstream signaling pathway may have arisen after the divergence of fish and tetrapods.

Although the TICAM ortholog has evolved in the basal chordate amphioxus, it does not induce the production of type I interfern (140). Actually, IRF3 and IRF7, the transcription factors essential for IFN1 production, have not been identified in phyla lower than the jawed cartilaginous fish. Hence, the antiviral interferon system only exists in vertebrates and evolved much later than the cytokine-producing NF-κB network, as the later already exists in Cnidaria.

## Conclusion

Toll-like receptors and their induced signaling networks play critical roles in development and innate immunity, and have been studied in detail in a broad range of organisms in the past decades. However, comparative studies regarding this tremendous signaling system and the evolutionary pattern scenario across Animalia are limited. In the current review, we have focused on the number, biological function, associated signaling pathways, and adaptor molecules of TLRs in non-mammals. The TLR repertoire in invertebrates and amphioxus is more abundant than that in vertebrates, which may be associated with the variation of lifecycles, lifetimes, or environments. Functionally, the vertebrate TLRs are involved specifically in immunity (except in amphibians), while the invertebrate TLRs also participate in development. A co-evolutionary pattern appears to exist between the MyD88–NF-κB signaling and TLR receptors ever since the first emergence of rudimentary TLR in Porifera. The TRIF-mediated TLR signaling evolved much later, as the first TRIF ortholog did not appear prior to the evolution of chordates and the TRIF-mediated production of interferon only exists in vertebrates. Despite different in TLR numbers, the TLR network appears to have been essentially conserved during evolution, especially regarding its structure and function in the vertebrates. However, ambiguities and gaps still exist in this field, as TLR-related studies are limited in some species of evolutionary importance (e.g., in Chondrichthyes and Reptilia), and thorough characterizations of the signaling adaptors, regulatory mechanisms, as well as crosstalk between different TLR signaling pathways are relatively rare in non-mammalian species. Hence, further systematic and integrated studies are expected to pave the way for the characterization of TLR networks across Animalia.

## Author Contributions

LN and S-YC collected all the reference publications for this review. LN wrote the sections of TLRs in invertebrates and amphioxus. LN, J-ZS, and JC wrote the section of TLRs in vertebrates and organized the paper. SY-C drew the tables.

## Conflict of Interest Statement

The authors declare that the research was conducted in the absence of any commercial or financial relationships that could be construed as a potential conflict of interest.
